# Extension of the TraPPE Force Field for Battery Electrolyte Solvents

**DOI:** 10.1021/acs.jpcb.2c06993

**Published:** 2023-03-02

**Authors:** Zhifen Luo, Stephen A. Burrows, Stoyan K. Smoukov, Xiaoli Fan, Edo S. Boek

**Affiliations:** † State Key Laboratory of Solidification Processing, School of Materials Science and Engineering, Northwestern Polytechnical University, 127 West Youyi Road, Xi‘an, Shaanxi 710072, People’s Republic of China; ‡ Chemical Engineering and Renewable Energy, School of Engineering and Materials Science, Queen Mary University of London, Mile End Road, London E1 4NS, United Kingdom

## Abstract

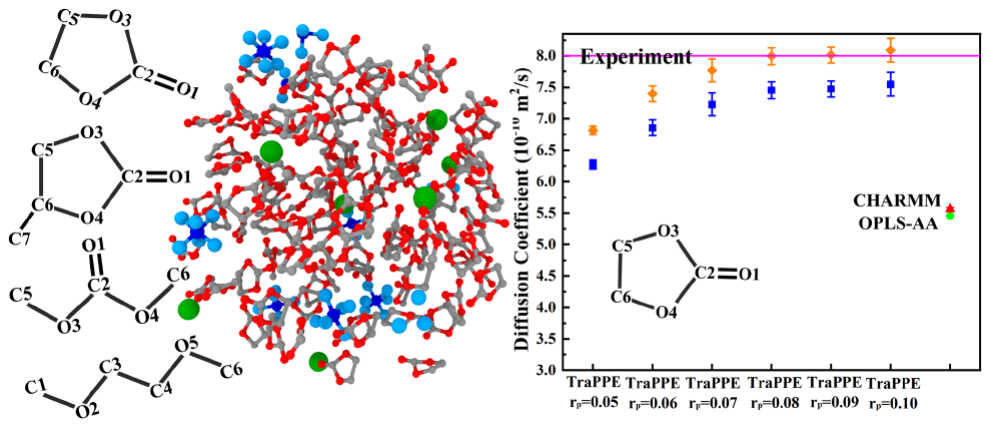

Optimizing electrolyte formulations is key to improving performance of Li-/Na-ion batteries, where transport properties (diffusion coefficient, viscosity) and permittivity need to be predicted as functions of temperature, salt concentration and solvent composition. More efficient and reliable simulation models are urgently needed, owing to the high cost of experimental methods and the lack of united-atom molecular dynamics force fields validated for electrolyte solvents. Here the computationally efficient TraPPE united-atom force field is extended to be compatible with carbonate solvents, optimizing the charges and dihedral potential. Computing the properties of electrolyte solvents, ethylene carbonate (EC), propylene carbonate (PC), dimethyl carbonate (DMC), diethyl carbonate (DEC), and dimethoxyethane (DME), we observe that the average absolute errors in the density, self-diffusion coefficient, permittivity, viscosity, and surface tension are approximately 15% of the corresponding experimental values. Results compare favorably to all-atom CHARMM and OPLS-AA force fields, offering computational performance improvement of at least 80%. We further use TraPPE to predict the structure and properties of LiPF_6_ salt in these solvents and their mixtures. EC and PC form complete solvation shells around Li^+^ ions, while the salt in DMC forms chain-like structures. In the poorest solvent, DME, LiPF_6_ forms globular clusters despite DME’s higher permittivity than DMC.

## Introduction

Lithium-ion batteries (LIBs)^1,2^ are currently widely exploited for high-performance electrochemical energy storage. LIBs have largely revolutionized our modern life^3−5^ and are widely applied in electric and hybrid vehicles.^6,7^ However, the time required to recharge LIBs for vehicle applications is still a bottleneck. Just as important for meeting CO_2_ reduction goals is utility-level battery storage, to smoothen the energy supply from intermittent renewables (solar, wind), with both high capacity and power. The Hornsdale Power Reserve in Australia delivering 150 MW (194 MWh) is an undeniable success, and utility battery storage is set for rapid growth.^8^ Current technologies and costs are available at NREL’s Annual Technology Baseline (ATB),^9^ but the development of better LIBs and future sodium-ion batteries with higher energy and power density as well as shorter recharging times are essential to improving our daily lives, especially for the widespread adoption of electric vehicles and utility storage. We briefly describe the structure of the batteries to highlight current challenges.

An LIB cell consists of two porous electrodes and an electrolyte. The two electrodes, normally a graphite anode and a transition metal oxide cathode,^10−12^ provide the host materials for Li^+^ intercalation. The electrolyte is usually composed of lithium salt dissolved in an ion-conductive solvent and acts as a separator to keep the two electrodes apart. The performance of LIBs depends strongly on the combination of the electrodes, salt, and solvent.^2^ The standard salt commonly used in Li^+^-ion batteries is LiPF_6_.

In LIBs, the electrolyte solution usually consists of high-permittivity cyclic carbonates including ethylene carbonate (EC) and propylene carbonate (PC), mixed with linear carbonates, such as dimethyl carbonate (DMC), diethyl carbonate (DEC), and ethyl methyl carbonate (EMC) .^13^ The choice of solvent is an important determining factor in the performance of the lithium-ion battery.^14,15^ Specifically, a good solvent prevents the formation of ion–ion pairs as they reduce the number of free charge-carrying ions, negatively affecting the conductivity.^15^ Not only are the ionic conductivity and transport properties of Li^+^ ions are affected by the properties of the solvent alone but also they change significantly as a function of salt activity. Hence salt and solvent choice are important to the overall performance, aging, and safety of the LIB,^16^ and the effect of the electrolyte composition on the physical properties of the solution, Li^+^ diffusion coefficient, and conductivity all need to be understood.

Commonly used methods to investigate the structure of the electrolyte and interactions of dissolved salt ions with solvent molecules include neutron diffraction measurements,^17,18^ nuclear magnetic resonance (NMR),^19−26^ and vibrational spectroscopies (infrared (IR) spectroscopy and Raman scattering)^18,19,22,24,27,28^ as well as computational methods: quantum chemical calculations^29,30^ and molecular dynamics (MD) simulations.^31−34^

Neutron diffraction has been used to determine the coordination number and nearest-neighbor distance of Li^+^ to the carbonyl oxygen in EC solvent.^18^ Neutron scattering experiments require synchrotron radiation facilities and therefore are not widely available. NMR is a commonly used method to accurately measure the diffusion coefficient of electrolyte components.^20^ For vibrational spectroscopies, the wide variety of sample types, smaller sample requirement, and fast analysis have enabled their widespread usage in structure and composition analysis.^35^ Raman spectroscopy has been used to determine conformer populations in 1,2-dimethoxyethane (DME),^36,37^ a widely used low-permittivity solvent in electrolytes. However, Raman may not be appropriate for all samples, as there are not only limitations due to sensitivity and absorption but also the issue of fluorescent emission of photons which interferes with the spectra from Raman scattering. This can be mitigated by using near-IR excitation, since the near-IR photons usually do not have enough energy to induce the excited states that cause fluorescence (see ref (35), p 32).

At a typical salt concentration of 1 mol/L (1M), the number of Li^+^ ions and counterions is at least 1 order of magnitude less than the number of solvent molecules. Hence the total number of molecules in the simulated electrolyte system must be at least thousands to obtain reasonable statistics for ionic properties. Larger systems are also desirable to mitigate finite-size effects associated with diffusion coefficient measurements.^38^ Because of the high computational cost of quantum chemistry simulations for systems of this size, MD simulation becomes the most viable choice to investigate the electrolyte properties. It can reproduce properties at conditions (e.g., temperature and pressure) difficult to access in experiment. By analyzing the coordination structure, MD simulation can also study the mechanism of Li^+^ diffusion. Carrier diffusion implies complete surrounding of Li^+^ by its solvent shell, which diffuses as a unit, whereas jump diffusion involves hopping of Li^+^ between solvent molecules.^15^

An ideal solvent should have a high polarity (relative permittivity ϵ > 15) in order to enable the full dissolution of the salt, since it can screen the ionic charges and decrease the attractive interactions between cations and anions more effectively. A low viscosity is also desirable to improve the mobility of Li^+^ ions, as is a wide operating temperature range^39,40^ and good safety profile such as high autoignition and flash point temperatures.^41^ Cyclic carbonates such as EC and PC are examples of aprotic solvents with high stability within a broad operating temperature range. Ethylene carbonate is the most widely used electrolyte solvent in LIBs because of its high relative permittivity of 90.5^42,43^ so that the salt will be well dispersed. The presence of EC and PF_6_^–^, the typical counterion of Li^+^, supports the formation of a stable solid electrolyte interface (SEI)^44^ on the surface of graphite. The main disadvantage of EC by itself is its somewhat higher viscosity, but it can achieve high ionic conductivity when mixed with low-permittivity additives, further increasing the performance.^45^ Another common high-permittivity solvent is PC, with a high dielectric constant of 64.92, but also high viscosity of 2.53 cP at 25 °C.^46^ Therefore, a suitable choice for formulating electrolytes will typically combine high-permittivity cyclic carbonates with low-viscosity linear carbonates, achieving optimal performance in LIBs.

The OPLS-AA force field^47^ has been optimized over many years for the simulation of liquids, using all-atom (AA) potentials. You et al. applied the OPLS-AA force field to EC and PC, investigating the dielectric constants, relaxation times, and molecular mobilities.^48^ The CHARMM all-atom force field^49,50^ is widely used for MD studies of biomolecules in particular, and the SwissParam tool also allows CHARMM input files to be generated for small organic molecules such as electrolyte solvents.^51^ Caleman et al.^52^ performed benchmark simulations for 146 organic liquids to compute their density, heat of vaporization, surface tension, compressibility, relative permittivity, and more, using OPLS-AA, generalized Amber force field (GAFF), and CHARMM. The results obtained for OPLS-AA and CHARMM appeared to be slightly better than those for GAFF for small organic molecules. Nunez Rojas et al.^53^ used the united-atom version of the transferable potentials for phase equilibria force field (TraPPE) to measure properties of 41 polar liquids, and discovered that the relative permittivity was typically underestimated but found a good overall level of accuracy for the density, heat of vaporization, and surface tension.

Force fields derived from OPLS-AA have been found to underestimate diffusion coefficients of both pure carbonate solvents^48^ and LiPF_6_ in solution.^54^ The many-body polarizable model of Borodin and Smith^55^ performs well but is significantly more computationally expensive, and the parameters are not publicly available. Chaudhari et al.^56^ reported that rescaling the charges of electrolyte solvent molecules in the OPLS-AA force field can achieve optimization of LiPF_6_ diffusion coefficients. However, they did not find a constant rescaling factor that worked well for all molecules, recommending factors of 80%, 90%, and 100% for EC, PC, and LiPF_6_, respectively. Earlier, a similar approach was applied to ionic liquids by Chaban.^57^ Karatrantos et al.^34^ achieved excellent prediction of PC, Li^+^, and PF_6_^–^ diffusion coefficients using the GAFF force field with PC charges scaled by 90% and ions by 85%.

In this work, we extend the computationally efficient united-atom TraPPE force field to support carbonate solvents, optimizing point charges for EC, PC, DMC, DEC, and DME. We note that DME is a linear ether rather than a carbonate and is already supported by TraPPE, but is included due to its wide use in LIBs. We measure the density, self-diffusion coefficient, permittivity, surface tension, and viscosity of some representative pure and mixed electrolyte solvent systems using the newly optimized TraPPE parameters. Using the OPLS-AA and CHARMM force fields as MD benchmarks, we compare the newly optimized TraPPE potential results with the corresponding experimental ones. We focus on lithium cation electrolytes with a hexafluorophosphate (PF_6_^–^) counterion, which is the most common choice of anion for Li-ion batteries.^46^

## Methods

### Software and Force Field

GROMACS^58,59^ version 2019.3, compiled in single precision, was used for all MD simulations in this work. Simulations were executed on Intel Xeon Gold 6248 CPUs.

The TraPPE^60^ united-atom force field was used as the foundation to develop an efficient MD model for electrolyte solvents. Suitable Lennard-Jones (LJ) parameters for carbonate solvents were chosen by identifying the most similar atom types from the ether,^60^ acrylate,^61^ and cyclic ether^62^ force fields. The LJ parameters chosen are reported alongside the corresponding optimized charges in Results and Discussion.

The TraPPE models for glycols^60^ and acrylates^61^ include intramolecular 1–4 Coulomb interactions (for atoms separated by three bonds) but scaled by a factor of 0.5, with 1–4 LJ interactions excluded. This convention is also adopted in this work. The LJ potential was truncated using a 1.4 nm cutoff, and the LJ tail correction to energy and pressure was used (DispCorr = EnerPres within the GROMACS.mdp file). All bond lengths were constrained, as is standard for TraPPE, which was done using the LINCS algorithm.^63^

LJ and charge parameters for LiPF_6_ are taken from OPLS-AA^64^ and are also used by the Canongia Lopes and Padua ionic liquid force field.^65^ These parameters are provided in Supporting Information Table S1.

**Figure 1:**
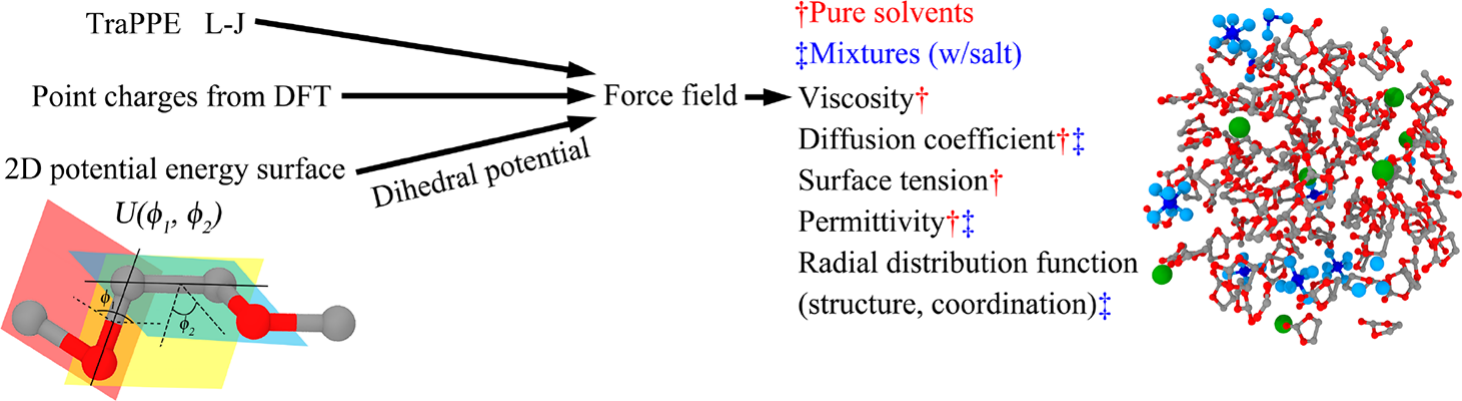
Overall strategy used to extend the TraPPE force field in this work.

The optimization scheme described in this work follows that of Burrows,^66^ in which TraPPE dihedral (torsion) potentials for EC, PC, DMC, and DME were fit to two-dimensional potential energy surfaces (PESs). We use these dihedral potentials unmodified in this work. A description of the optimization algorithm and contour plots of the PESs are provided in the Supporting Information, as are complete force field files for our GROMACS implementation. The overall strategy for the optimization is illustrated in Figure 1.

### Charge Optimization

To calculate point charges for the new molecules, density functional theory (DFT) calculations were performed using the B3LYP hybrid functional^67−69^ with Grimme dispersion correction D3^70^ and the aug-cc-pvtz basis set.^71,72^ The NWChem software^73^ was used for all DFT calculations. The electrostatic potential (ESP) fitting module was used to obtain charges which best reproduce the ESP around the molecule. As this is a united-atom model, the charges on all hydrogen atoms were constrained to zero so that point charges are optimized at the united-atom sites only. The grid points at which the ESP is computed are located in an envelope surrounding the molecule, defined by the region outside the probe radius *r*_p_ but within the cutoff distance *r*_max_ from the nuclei positions. Therefore, reducing the *r*_p_ value means this envelope approaches the nuclei more closely. However, since the purpose of the charges is to model intermolecular Coulomb interactions, fitting the ESP very close to the nucleus is less important. In this work, a range of *r*_p_ values are tested from 0.05 to 0.10 nm, with NWChem suggesting a default value of 0.07 nm. *r*_max_ was set to the default value of 0.3 nm. The ESP grid spacing was set to 0.01 nm.

### Simulation Setup

All simulations of liquids begin with energy minimization followed by a 4 ns equilibration phase in the *NPT* ensemble using the Berendsen barostat^74^ with a time constant of 1 ps and compressibility of 5 × 10^–5^ bar^–1^. After this, a 12 ns *NPT* data collection simulation is carried out using the Parrinello–Rahman barostat.^75^ The Bussi–Donadio–Parrinello thermostat,^76^ denoted v-rescale in GROMACS, was used to control the temperature in all simulations with a time constant of 0.1 ps. The number of molecules was chosen to target a cubic simulation box size of ≈8 nm, and the system used periodic boundary conditions with the Ewald summation for long-range electrostatics.

For all reported properties, we obtained results averaged over 5 independent runs, each with a different random seed to generate the initial atom velocities at the start of the equilibration phase. In order to match the temperature used in the diffusion experiments by Hayamizu et al.,^20^ simulations of pure PC, DMC and DME were performed at 303 K, with 313 K being used for EC due to its higher melting point. Simulations of electrolyte systems with salt were performed at 298 K. The pressure was set to 1 bar for all systems. Systems containing salt used a 1 M (1 mol/L) concentration of LiPF_6_. The numbers of solvent and salt molecules for each system are tabulated in the Supporting Information.

### Property Measurement

#### Diffusion Coefficient

The diffusion coefficient was computed from the mean squared displacement (MSD = ⟨|**r**_*i*_(*t*) – **r**_*i*_(0)|^2^⟩), using the Einstein relation
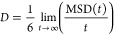
1where *D* is the self-diffusion coefficient, **r**_*i*_(*t*) is the position vector of atom *i* at time *t*, and ⟨⟩ represents the average over all atoms. MSD was obtained from the 12 ns *NPT* simulation by a linear fit. The finite-size correction of Yeh and Hummer^38^ was applied to the self-diffusion coefficients measured for pure solvents,
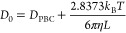
2where *D*_0_ is the corrected value, *D*_PBC_ is the value measured by mean squared displacement in a periodic box of length *L*, and the viscosity η was computed as described below. This correction was not applied to compute diffusion coefficients of systems containing salt, as it was validated for single-component Newtonian fluids.

#### Relative Permittivity

The relative permittivity, ε, was calculated from fluctuations of the total dipole moment of the system using the GROMACS tool *gmx dipoles*, which uses the formula^77−79^
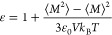
3where ε_0_ is the vacuum permittivity, *V* is the volume of the simulation box, *T* is the constant simulation temperature, *k*_B_ is the Boltzmann constant, *M* denotes the total dipole moment of the simulation box, and ⟨⟩ represents the time average.

#### Surface Tension

The surface tension, γ, is derived from the difference in average pressure between the *z* direction, which is normal to the interface, and the in-plane *x* and *y* directions. GROMACS obtains the average pressure components by integration, and therefore γ is defined as^80^
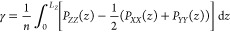
4where *L*_*Z*_ is the length of the simulation cell in the *z* direction and *P*_*XX*_, *P*_*YY*_, and *P*_*ZZ*_ are the three pressure components along the *x*, *y*, and *z* directions, respectively. *n* is the number of interfaces, which is two in our periodic system. In this work, an equilibrated *NPT* system with isotropic pressure coupling was taken to get the starting configuration for the liquid phase region. Then, we enlarged the simulation box by a factor of 3 in the *z* direction to create the interface, and the simulation was performed in the *NVT* ensemble to obtain the vapor–liquid surface tension. For surface tension measurements only, the LJ cutoff was increased to 2.5 nm to mitigate truncation effects.^81^

### Viscosity

The viscosity can be calculated from a non-equilibrium simulation using the cosine-acceleration method.^82^ In this approach, a spatially varying acceleration is applied to the atoms, with the following cosine form
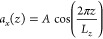
5where *A* is a parameter which specifies the maximum acceleration. Assuming a Newtonian fluid, the generated velocity profile *v*_*x*_(*z*) will have the form
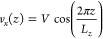
6After fitting this cosine velocity profile and obtaining the maximum velocity *V*, the viscosity is computed from *A* and *V* by
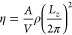
7where *A* is the cos-acceleration parameter in the GROMACS.mdp file. In Supporting Information Figure S5, the calculated viscosity is presented as a function of *A*. We see the viscosity decreases as *A* increases, which corresponds to increasing shear rate. Based on the extrapolation shown in Figure S1, a cos-acceleration parameter of *A* = 0.003 nm/ps^2^ results in a computed viscosity close to the zero shear rate result obtained by extrapolation to *A* = 0. Therefore, this value was chosen for all of the solvents when measuring viscosity.

### Coordination Number

The radial distribution function (RDF) provides a basis for short-range structure analysis at the atomic level. It is a measure of the probability of finding particles of type *j* around particles type of *i* at distance *r*.

The RDF was determined by calculating the distance between all pairs of particles *i* and *j* and then producing a histogram of these values. The histogram is then normalized with respect to the number density of the type *j* particles, ρ_*j*_, multiplied by the volume of the spherical shell between radii *r* and *r* + d*r*, which can be expressed as ρ_*j*_4*πr*^2^ d*r*, where d*r* is the size of the histogram bin. The RDF, *g*_*ij*_(*r*), is therefore given by
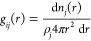
8where d*n*_*j*_(*r*) is the mean number of type *j* particles in a shell at *r* of width d*r*.

The coordination number *N*(*r*_c_) is calculated from the RDF, as
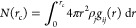
9where *g*_*ij*_(*r*) is the RDF for particles of type *i* and *j* and ρ_*j*_ is the average number density of type *j* particles. Integrating up to a cutoff, which in this work was set to *r*_c_ = 4.5 Å, yields the average number of type *j* particles in the first coordination shell around type *i*.

### DMC Conformers

DMC has two stable conformers with a large energy barrier separating them and may therefore require long simulation times to reach the equilibrium distribution. To investigate this, we perform simulations with DMC starting in each of these conformers, denoted str1 (cis–cis) and str2 (cis–trans), as shown in Figure S6. In str1, both O=C–O–CH_3_ dihedrals are in the cis conformation. We use the time evolution of the RDF for the distance between atoms O1 and C5/C6 to check the final equilibration time, since the O1–C5/C6 interatomic distance varies between the two conformers. After 14 ns of simulation starting from str1, only a very small peak appears corresponding to the O1–C5/C6 distance of str2 at 0.34 nm, indicating the vast majority of the molecules remain in str1. When starting with all molecules in str2, we observe the peak corresponding to str2 decreasing continually and still decreasing at the end of a 24 ns simulation. These results suggest str1 has significantly lower energy than str2. Hence, we carried out DFT energy calculations of the two conformers to confirm this and to estimate their populations, using the same functional and basis set as described in Charge Optimization. The equilibrium fraction of str1 was estimated by
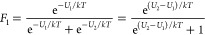
10where *F*_1_ is the fraction of str1 and *U*_1_ and *U*_2_ are the energies of str1 and str2, respectively. We find the energy difference *U*_2_ – *U*_1_ to have a value of 11.829 kJ/mol, resulting in the estimated fraction of conformer str1 being approximately 0.99, which defines the starting ratio of conformers used in DMC simulations. However, this can only be taken as a guideline since the DFT energy computation is performed in vacuum and ignores intermolecular effects which may stabilize certain conformers. Previous estimates of the str1 population are in the range 0.94–0.98.^83,84^

## Results and Discussion

### Force Field Optimization and Pure Solvent Properties

**Figure 2:**
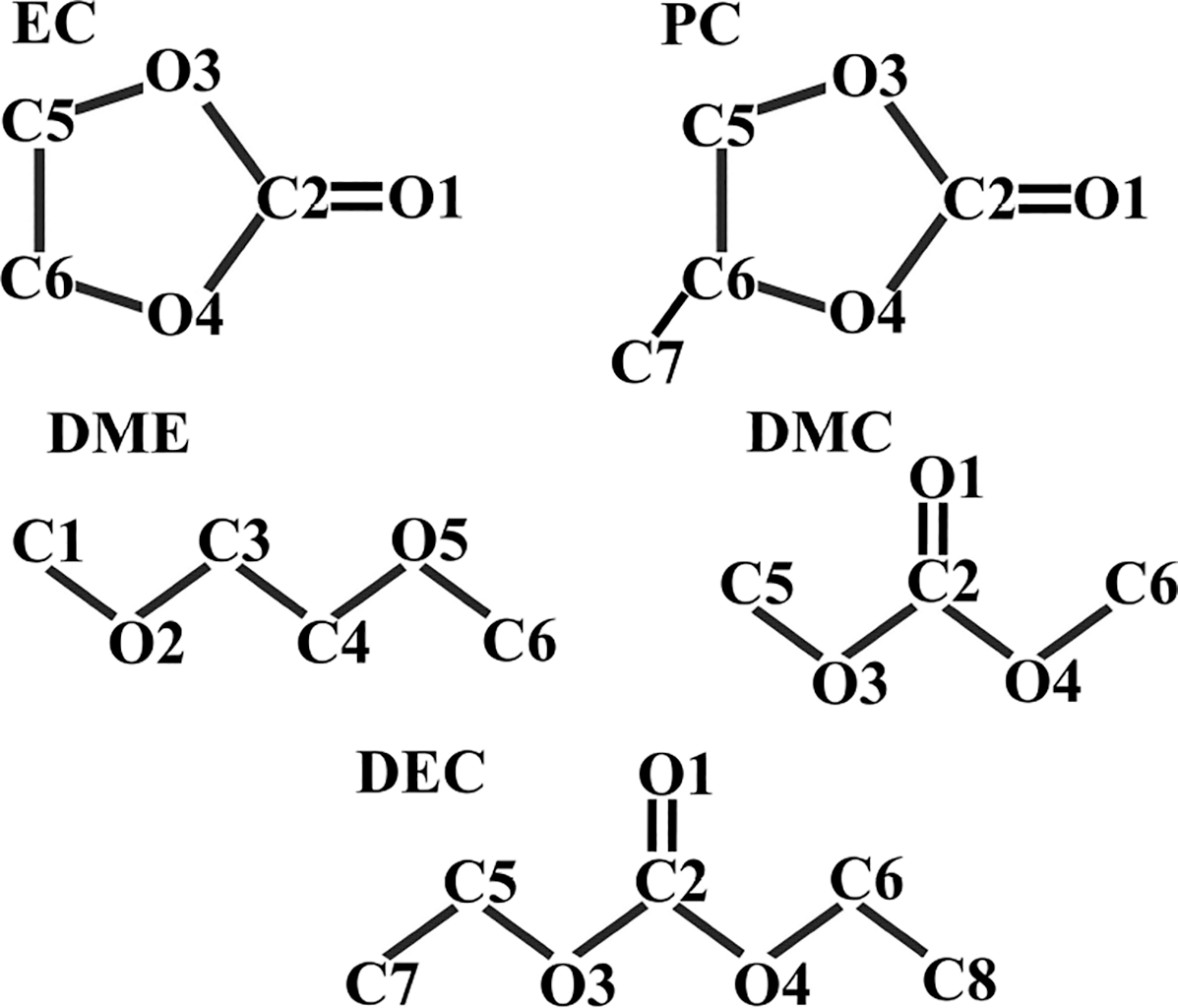
Chemical structures of electrolyte solvents with abbreviations and atom numbers.

First, NWChem^73^ was used to optimize the atomic charges for the molecules EC, PC, DME, DMC, and DEC. Figure 2 shows the chemical structure of these five solvents with atom numbers. In Figure S7, the optimized charges for different values of *r*_p_ are reported to illustrate the sensitivity of the charges to this variable. The definition of *r*_p_ and the DFT settings are explained in Charge Optimization. As expected, the oxygen atoms are assigned negative charges, with the carbonyl oxygen (O1) charge being largest in magnitude. The absolute charges of double-bonded O1 and the connected C2 atoms decrease as the parameter *r*_p_ is increased from 0.05 to 0.1 nm for EC, PC, DMC, and DEC. However, for the DME solvent, the point charges of the terminal C1/C6 atoms are observed to increase, while the charges of central C3/C4 atoms decrease, as the *r*_p_ value is increased.

Then we used the TraPPE force field with different candidate sets of atomic charges (corresponding to different values of *r*_p_), alongside the all-atom force field CHARMM, to measure the self-diffusion coefficient and relative permittivity of pure EC, PC, DMC, DEC, and DME solvents. Input files for the CHARMM all-atom force field were generated using the SwissParam tool^51^ (denoted CHARMM-SP) which is designed for small organic molecules. The dependence of self-diffusion coefficients and relative permittivities of the pure liquids EC, PC, DMC, and DEC on *r*_p_ are presented in Figures S8 and S9. All-atom OPLS-AA results for the diffusion coefficient and relative permittivity of EC and PC were obtained by You et al.^48^ and are used as an additional benchmark for those molecules.

Using the data in Figures S8 and S9, we compare the predictions of the self-diffusion coefficient and relative permittivity to choose the most suitable atomic charges for the extended TraPPE force field. The charge set corresponding to *r*_p_ = 0.08 had the highest level of accuracy in predicting the diffusion coefficient of the carbonate solvents (EC, PC, and DMC) after applying the finite size correction proposed by Yeh and Hummer et al. .^38^ As we prioritize transport properties, we select this charge set. However, slightly more accurate prediction of relative permittivity was obtained using *r*_p_ = 0.1. The numerical values of the selected charges, computed with *r*_p_ = 0.08, are reported in Table 1 alongside the corresponding LJ parameters. The same parameters using TraPPE’s standard units of Å and K are provided in Supporting Information Table S2.

**Table 1:** LJ Parameters from the TraPPE Force Field^60−62^ and Newly Optimized Charges, Where σ and ϵ Are the LJ Size Parameter and Well Depth, Respectively

Molecule	Atom	σ (nm)	ϵ (kJ/mol)	Charge (|*e*|)
EC	O1	0.305	0.6568	–0.547
	C2	0.382	0.3326	0.825
	O3, O4^a^	0.220	1.5797	–0.399
	C5, C6 (CH_2_)^a^	0.388	0.4681	0.260
PC	O1	0.305	0.6568	–0.547
	C2	0.382	0.3326	0.825
	O3, O4^a^	0.220	1.5797	–0.399
	C5 (CH_2_)^a^	0.388	0.4681	0.260
	C6 (CH)	0.433	0.0831	0.260
	C7 (CH_3_)	0.375	0.8148	0.0
DMC	O1	0.305	0.6568	–0.614
	C2	0.382	0.3326	0.932
	O3, O4	0.280	0.4573	–0.448
	C5, C6 (CH_3_)	0.375	0.8148	0.289
DEC	O1	0.305	0.6568	–0.614
	C2	0.382	0.3326	0.932
	O3, O4	0.280	0.4573	–0.448
	C5, C6 (CH_2_)	0.395	0.3825	0.289
	C7, C8 (CH_3_)	0.375	0.8148	0.0
DME	C1, C6 (CH_3_)	0.375	0.8148	0.204
	O2, O5	0.280	0.4573	–0.428
	C3, C4 (CH_2_)	0.395	0.3825	0.224

a LJ parameters from ref (62) (five-membered cyclic ether). Others from refs (60) and (61).

The diffusion coefficients predicted by the TraPPE potential for the carbonate solvents are more accurate than those of CHARMM-SP, but for DME, the CHARMM-SP results are closer to the experimental values. We also compare the DME results to those using default TraPPE charges, in which oxygen and carbon have charges of −0.5 |*e*| and 0.25 |*e*|, respectively, finding the default force field more accurately reproduces the diffusion coefficient at the cost of a significant error in the relative permittivity. In addition, please note recent reparametrization of the TraPPE-UA model for oligoethers^85^ recommending a partial charge of −0.44 for the ether oxygen, i.e., closer to the charge of −0.4278 obtained in the current work. We provide both versions of the DME model in the Supporting Information input files.

The computed relative permittivity, ε, is shown in Table 2, with the dependence on *r*_p_ shown in Figure S9. We find that the TraPPE force field results of PC and DME correspond well to the experimental data. All force fields show some inaccuracy in reproducing ϵ for EC, with TraPPE and OPLS-AA overestimating ϵ by ≈30% and CHARMM-SP underestimating by ≈22%. As for DMC, the relative permittivities calculated via the TraPPE and CHARMM-SP potential are both approximately 50% below the experimental value, with TraPPE being slightly more accurate. The DMC solvent has two possible conformers, each with a different dipole moment, and therefore the ratio of these two conformers may influence the measured permittivity. The cis–trans conformer, denoted str2 in Figure S6, has a larger dipole moment,^83^ and therefore the underestimation of ε may arise from an underestimation of this conformer’s population.

**Table 2:** Computed Densities (ρ), Diffusion Coefficients (*D*), Relative Permittivities (ε), Surface Tensions (γ), and Viscosities (η) of EC (313 K), PC (303 K), DMC (303 K), DEC (298 K), and DME (303 K) Using the TraPPE Force Field

	Solvent	Computed value ± SD	Experimental data	Relative errors^k^ (%)
ρ (g/cm^3^)	EC	1.330^j^	1.323^a^	0.49
	PC	1.219	1.200^b^	1.56
	DMC	1.064	1.063^b^	0.04
	DEC	0.973	0.980^c^	–0.71
	DME	0.834	0.860^b^	–3.02
	DME def-q^l^	0.863	0.860^b^	0.35
*D* (10^–10^ m^2^/s)	EC	7.996 ± 0.135	8.00^d^	–0.05
	PC	5.886 ± 0.073	5.80^d^	1.48
	DMC	25.918 ± 0.457	26.00^d^	–0.32
	DEC	23.360 ± 0.457	-	-
	DME	51.217 ± 1.214	31.00^d^	65.22
	DME def-q^l^	41.438 ± 1.413	31.00^d^	33.67
ε	EC	118.546 ± 3.550	89.00^a^	33.20
	PC	77.272 ± 7.53	64.90^e^	19.06
	DMC	1.616 ± 0.034	3.1^e^	–47.87
	DEC	1.372 ± 0.020	2.80^c^	–50.99
	DME	8.641 ± 0.202	7.20^e^	20.02
	DME def-q^l^	14.405 ± 0.149	7.20^e^	100.00
γ (mN/m)	EC	62.527 ± 2.036	50.60^a^	23.57
	PC	48.277 ± 1.167	40.5^i^	19.20
	DMC	34.278 ± 0.826	29.90^f^	14.64
	DEC	28.676 ± 0.465	26.3^g^	9.03
	DME	21.631 ± 0.967	24.70^g^	–12.43
	DME def-q^l^	25.167 ± 0.837	24.70^g^	1.89
η (mPa s)	EC	1.873 ± 0.066	1.90^a^	–1.41
	PC	2.496 ± 0.136	2.53^b^	–1.34
	DMC	0.603 ± 0.014	0.59^b^	5.81
	DEC	0.575 ± 0.019	0.61^c^	–5.70
	DME	0.263 ± 0.016	0.39^h^	–32.54
	DME def-q^l^	0.350 ± 0.004	0.39^h^	–10.18

a Experimental data from ref (86).

b Experimental data from ref (46).

c Experimental data from ref (87).

d Experimental data from ref (20).

e Experimental data from ref (46).

f Experimental data from ref (88).

g Experimental data from ref (89).

h Experimental data from ref (90).

i Experimental data from ref (91) (25 °C).

j For ρ, all standard deviations (SD) are <10^–4^ g/cm^3^ and therefore not shown.

k Relative errors are in comparison to experimental values.

l def-q refers to using default TraPPE charges (*q*_O_ = −0.5; *q*_C_ = 0.25|*e*|) with the newly optimized dihedral potentials.

To further validate the force field, we calculate the density, liquid–vapor surface tension and viscosity of pure EC, PC, DMC, and DME solvents with the TraPPE force field using the parameters given in Table 1 and compare them with the corresponding experimental values. The results are shown in Table 2. All of the density results agree with the experimental data well, as the errors are all ≤3%. The viscosity predictions for EC, PC, DMC, and DEC agree well with the corresponding experimental values, with their errors all being less than 6% in magnitude. The surface tension of the carbonate solvents is generally overestimated, by factors of 9% to 24%. For DME, we find the surface tension is reproduced to within 12% of the experimental value and only 2% when using the default charges. However, the error in the computed viscosity of DME is close to −33%.

The results in Table 2 show the default TraPPE charges of DME, which better reproduce the diffusion coefficient, are also better suited to reproduce DME’s viscosity (and surface tension). This is consistent with the Stokes–Einstein relation between diffusion coefficient and viscosity,
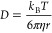
11where *k*_B_ is Boltzmann’s constant and *r* the hydrodynamic radius. If we assume *r* is mostly determined by the molecular size and not the charges, it is expected that a force field overestimating *D* will underestimate η. The default DME charges are larger in magnitude, so it is expected *D* will be lower, as larger charges increase the activation energy for diffusive motion.^57^ However, use of the default charges comes at the cost of overestimating the relative permittivity by a factor of 2. This overestimation of permittivity may be related to an increased concentration of DME’s most polar conformations, since liquids with larger molecular dipoles have higher permittivity.^46^ It is known that the TraPPE model of DME does not reproduce its conformer populations very accurately^66,92^ (please note, ref (92) has a published correction). In particular, TraPPE overestimates the population of the TGG conformer which has the largest dipole.^36^

To address the potential issue of statistical error, we obtain the standard deviation (SD) of the density, surface tension, and viscosity measurements from five independent simulations. For all molecules, the SD is less than 10%, with the SD being negligible for the density measurements. Hence, we conclude the extended united-atom TraPPE force field is suitable to describe the physical properties of solvents relevant to LIBs, especially in reproducing the transport properties of carbonates EC, PC, and DMC. However, the relative permittivity of DMC and DEC could not be reproduced accurately with either force field (TraPPE and CHARMM).

### Single Solvent and Salt Structure

**Figure 3:**
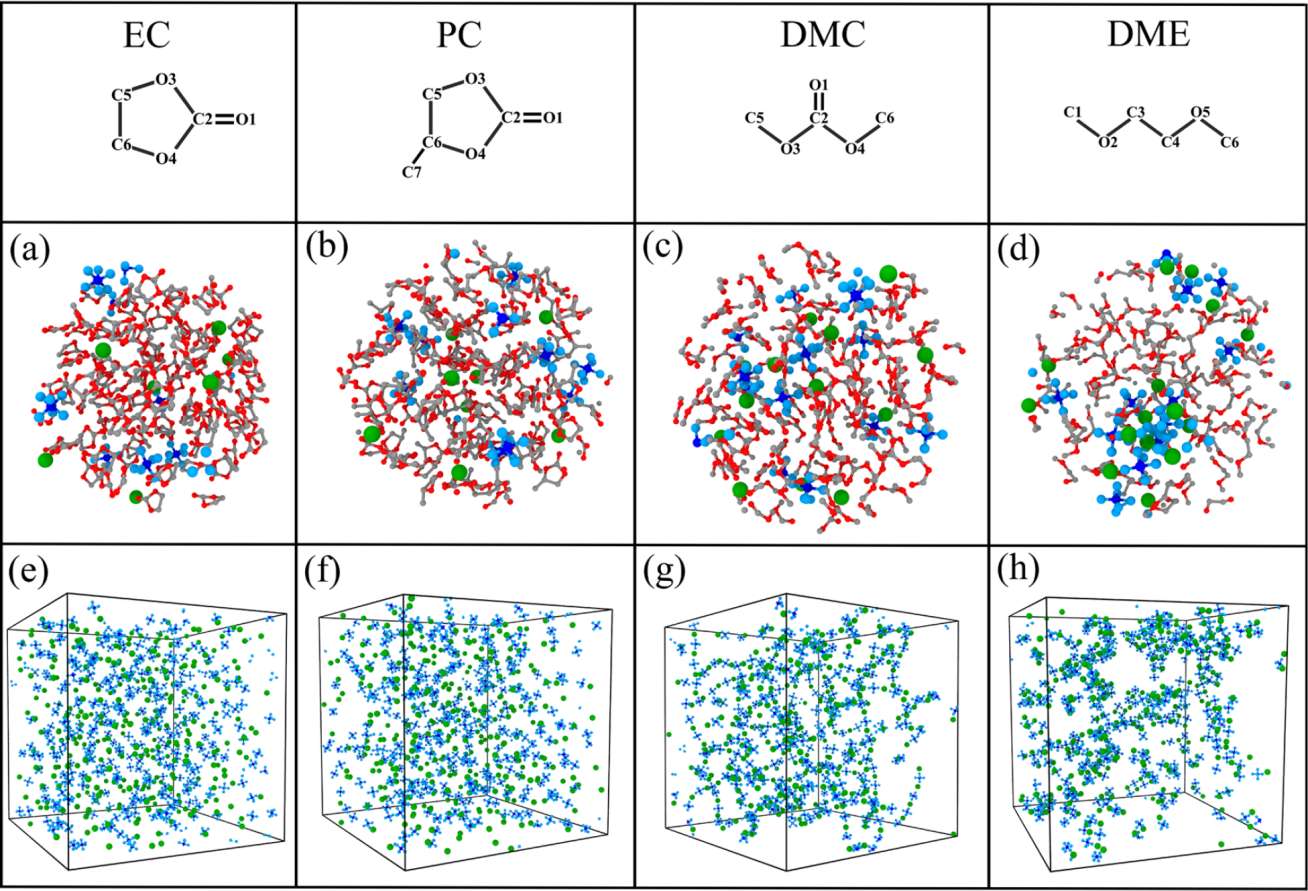
Final solvation structure and salt distribution of different single solvents with 1 M LiPF_6_ salt at 298 K, visualized using OVITO:^93^ (a) structure of EC with LiPF_6_; (b) structure of PC with LiPF_6_; (c) structure of DMC with LiPF_6_; (d) structure of DME with LiPF_6_; (e) distribution of LiPF_6_ in EC solvent; (f) distribution of LiPF_6_ in PC solvent; (g) distribution of LiPF_6_ in DMC solvent; (h) distribution of LiPF_6_ in DME solvent. The red, gray, green, and blue spheres represent O, C, Li^+^, and PF_6_^–^ atoms, respectively.

Figure 3 demonstrates the equilibrium solvation structure of different single solvents with 1 M LiPF_6_ and their salt distribution. In Figure 3a,b, after equilibration, the LiPF_6_ ions are distributed quite uniformly in the high-permittivity cyclic carbonates EC and PC. Despite having the lowest permittivity, DMC is found to distribute the salt more uniformly than DME, demonstrating the importance of the carbonyl oxygen in solvating small cations. In Figure 3g, the Li^+^ and PF_6_^–^ ions solvated by DMC are found to join together to form long chains of alternating positive and negative ions.

**Figure 4:**
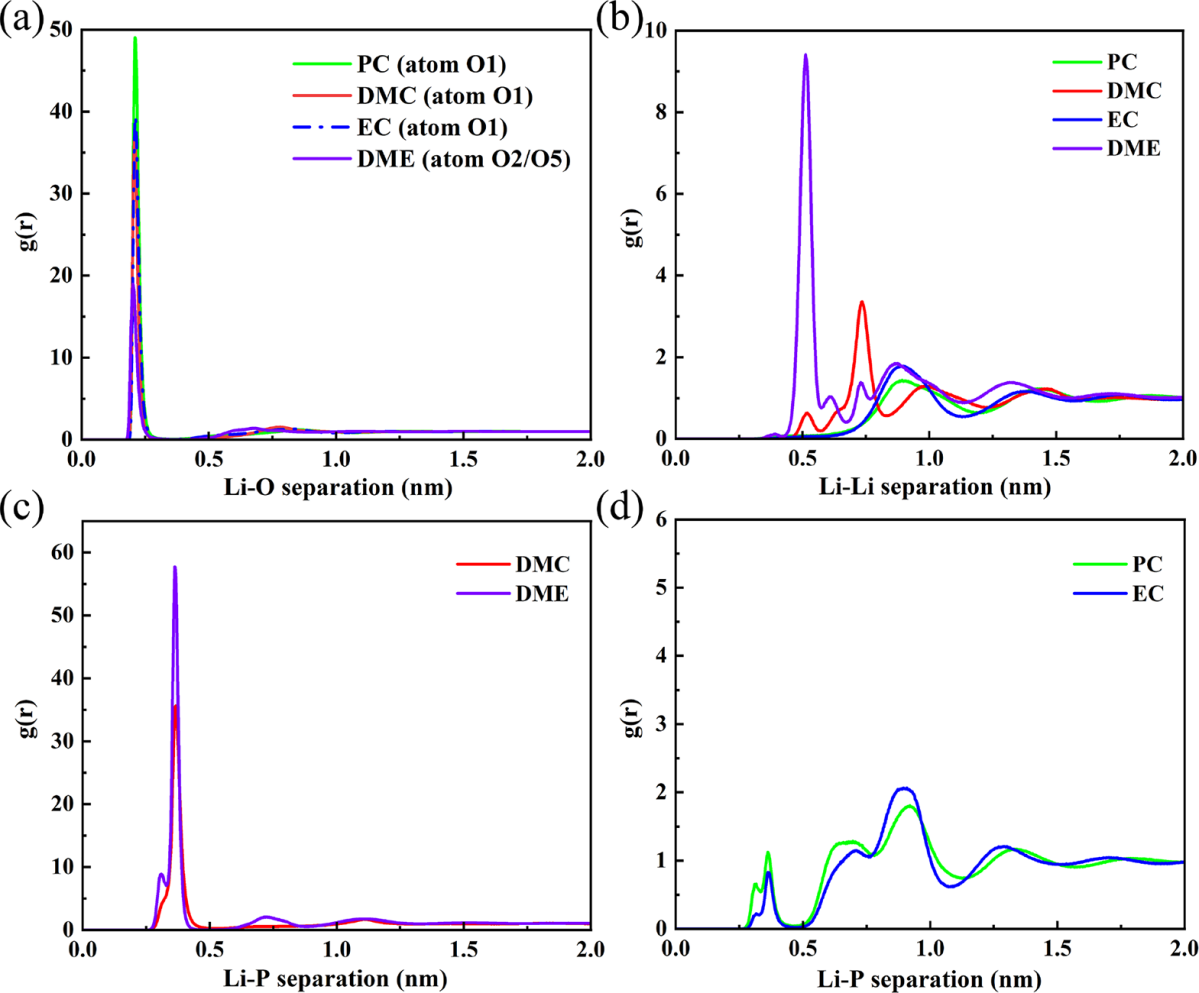
RDF of Li^+^ atoms with their surrounding O, Li^+^, and P atoms when dissolved in EC, PC, DME, and DMC pure solvents. (a) RDF of Li–O; (b) RDF of Li–Li; (c) RDF of Li–P with DMC and DME solvents; (d) RDF of Li–P with EC and PC solvents, all measured at 298 K.

These differences in structure can be quantified using the RDF, *g*(*r*), which describes radial fluctuations in the density around a given central atom. This is used to measure the coordination number of various pairs of atoms or molecules, such as the average number of PF_6_^–^ anions surrounding each Li^+^. We analyzed the RDF of Li^+^ ions with the neighboring atoms, O1 (carbonyl oxygen), Li^+^, and P, with the results being presented in Figure 4. The RDFs were calculated and time-averaged over the entire 12 ns trajectory, which contains 3000 frames. There is a large peak located at ≈0.25 nm in *g*(*r*) of Li^+^ with the double-bonded O1 in EC/PC/DMC solvents, indicating that there is a high density of carbonyl oxygens surrounding Li^+^ ions. Meanwhile, a smaller equivalent peak appears in the *g*(*r*) of Li^+^ with O atoms in the DME solvent. In the saline DME system, there is a strong peak in the RDFs of Li^+^ ions with both P and Li^+^ atoms at *r* ≈ 0.4 and 0.5 nm, respectively. This demonstrates incomplete dissolution of LiPF_6_, consistent with the cluster structures observed in Figure 3h. Comparing Figure 4c,d, the first peak in the Li–P RDF is far larger in DMC/DME than EC/PC.

**Figure 5:**
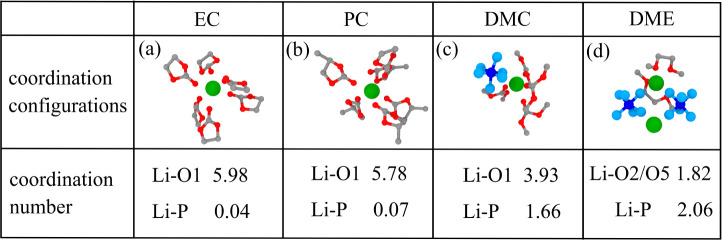
Visualization of nearest-neighbor solvation shells around a Li^+^ ion in solvents (a) EC, (b) PC, (c) DMC, and (d) DME. Coordination numbers for Li–O and Li–P pairs at 298 K are derived from integration of the corresponding RDF using a cutoff of 4.5 Å.

Then, we visualize the solvation shell around a Li^+^ ion in different solvents and compute the corresponding coordination number in Figure 5. EC and PC are highly polar, and the negatively charged carbonyl oxygen (O1) is located at the narrow pointed end of the molecule, which results in EC and PC forming a more complete solvation shell around Li^+^ than DMC and DME. We also integrate their corresponding RDF from 0 to 0.45 nm to derive the coordination number for Li–O (lithium—solvent) and Li–P (lithium—anion) pairs, with the results (also in Figure 5) consistent with the visualized configurations. For EC and PC, the number of O1 atoms in the Li^+^ solvation shell are both approximately 6, whereas the numbers of O atoms surrounding Li^+^ in DMC and DME solvents are about 4 and 2, respectively. There is negligible PF_6_^–^ located in the first solvation shell of Li^+^ in EC and PC solvents, which agrees well with the Li^+^-EC coordination number of 5.69 obtained by an NMR study of 1 M LiPF_6_ in pure EC solvent.^94^ In the saline DMC and DME systems, on the other hand, there are 1.8 and 2.1 PF_6_^–^ cations around the Li^+^ ions, respectively.

### Mixed Solvent Physical Properties

**Figure 6:**
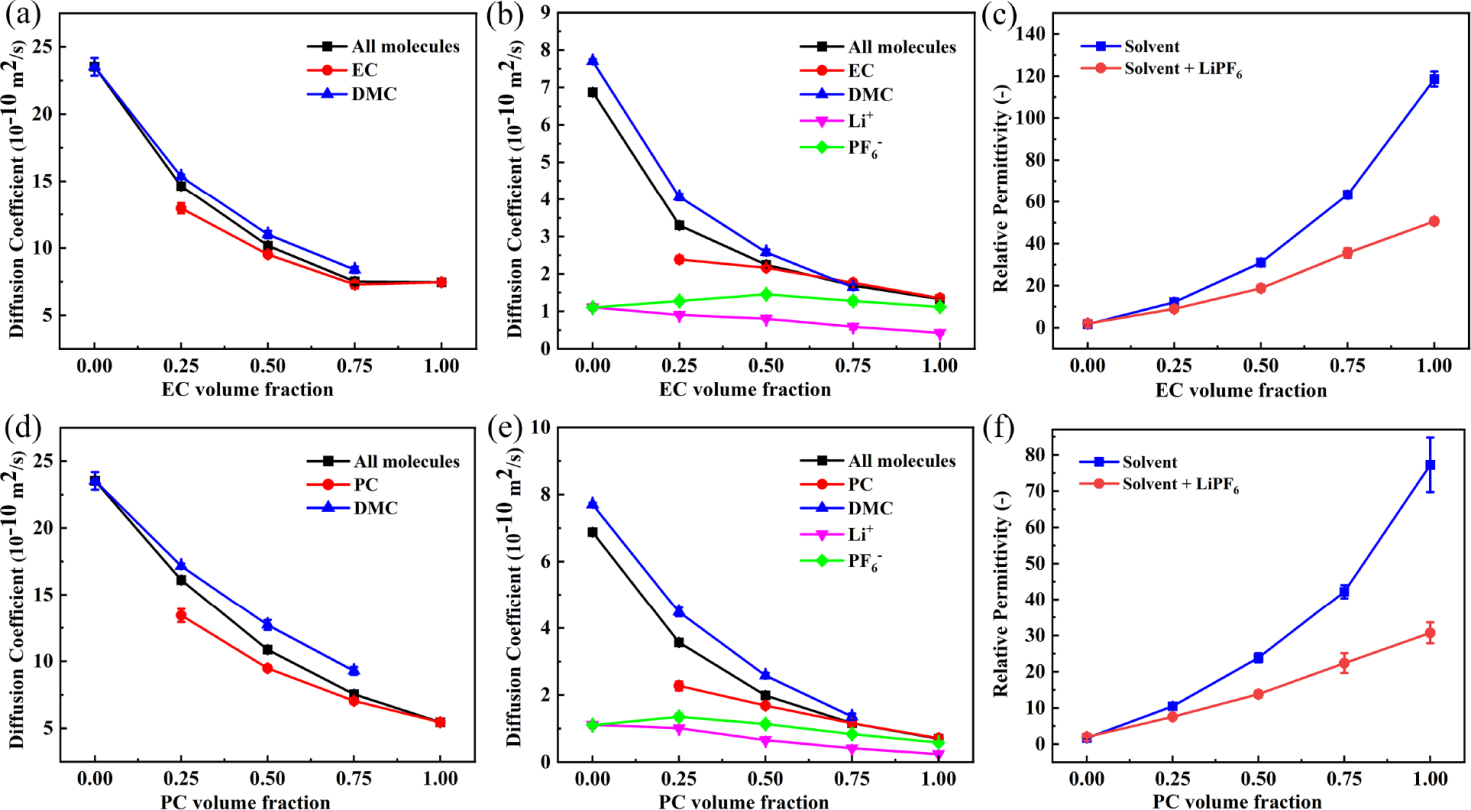
Computed diffusion coefficients and relative permittivities of binary solvent mixtures, some with 1 M LiPF_6_ (298 K): (a) diffusion coefficients of EC-DMC solvent mixture without salt; (b) diffusion coefficients of EC-DMC solvent mixture with 1 M LiPF_6_; (c) permittivities of EC-DMC solvent mixture both with and without salt; (d) diffusion coefficients of PC-DMC solvent mixture without salt; (e) diffusion coefficients of PC-DMC solvent mixture with 1 M LiPF_6_; (f) permittivities of PC-DMC solvent mixture both with and without salt.

We perform simulations using the extended TraPPE force field to measure self-diffusion coefficients and relative permittivities for relevant binary electrolytes (EC-DMC, PC-DMC) with varying volume fractions. Panels a and d of Figure 6 show that, as the EC or PC volume fraction is increased, the self-diffusion coefficient of all solvent molecules decreases sharply. After adding 1 M LiPF_6_ salt, the self-diffusion coefficient of all electrolytes is reduced by >50%, but the trend of the self-diffusion coefficient with EC/PC concentration stays the same for every component. The self-diffusion coefficients of PC and EC are always less than DMC in the solvent mixture with 1 M LiPF_6_. Since Li^+^ is the slowest diffusing species, this may indicate a greater fraction of EC and PC molecules are bound to Li^+^ as expected from the coordination analysis. In pure DMC, we see the diffusion coefficients of Li^+^ and PF_6_^–^ are nearly identical. This corresponds with the chain-like structures in Figure 3g, in which most ions are bound to counterions and therefore diffuse at the same speed. Unlike Li^+^ and the solvent molecules, the diffusion coefficient of PF_6_^–^ does not decrease monotonically as the EC or PC volume fractions are increased.

Compared to the all-atom MD and experimental data of Takeuchi et al.^54^ for 1 M LiPF_6_ in PC, the TraPPE results represent an improvement on their MD results which underestimate diffusion coefficients by a larger factor. However, as the diffusion coefficient is still too low, we investigate the uniform charge-rescaling approach applied by Chaban^57^ to ionic liquids. In this approach, the point charges of Li^+^ and PF_6_^–^ atoms are rescaled by a constant factor which is optimized to reproduce transport properties.

**Figure 7:**
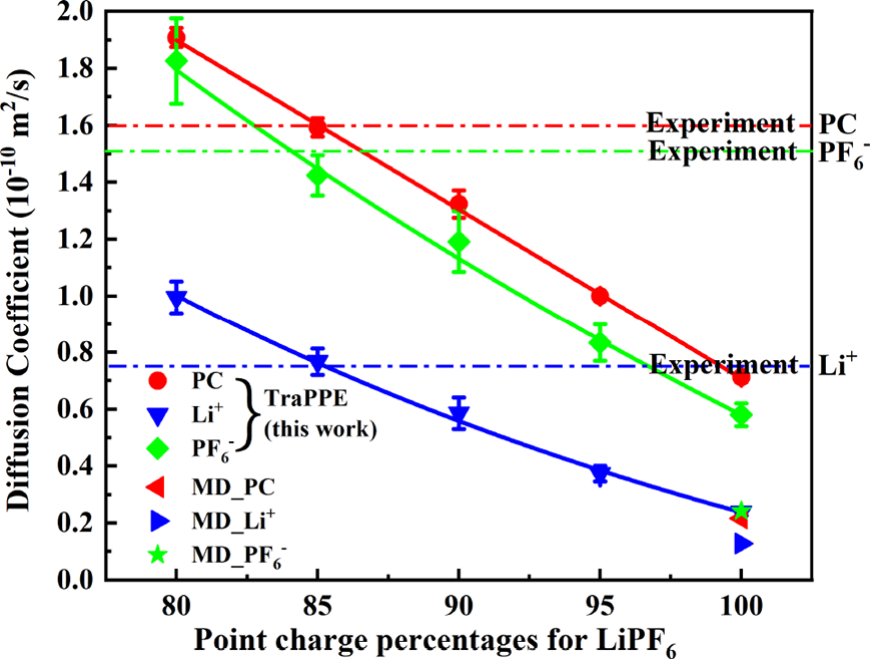
Diffusion coefficients of Li^+^, PF_6_^–^, and PC for 1 M LiPF_6_ in PC system (298 K). Charges of Li^+^ and PF_6_^–^ atoms are rescaled by factors from 80% to 100% (*x* axis). Experimental results are from Takeuchi et al.^54^ The given all-atom MD results (◀, ▶, ★) are also are from ref (54).

Figure 7 shows the dependence of the diffusion coefficients on the charge rescaling factor, finding a very strong dependence and suggesting an optimal factor of ≈85%. The errors compared to experimental data^54^ are −0.39%, 2.21%, and −5.67% for PC, Li^+^, and PF_6_^–^, respectively. We also find the TraPPE model reproduces the ratio of the diffusion coefficients for the different molecules very well, with PF_6_^–^ being slightly lower than PC and Li^+^ approximately half. At 85% charge rescaling, the Li^+^-O1 coordination number is reduced from 5.78 (Figure 5) to 5.13, which more closely aligns with values of ≈4.5 obtained from some neutron diffraction^17^ and NMR diffusion^22^ measurements.

Additionally, simulations using an 85% LiPF_6_ charge-rescaling factor were performed for a mixed electrolyte with EC:DMC at 50:50 wt % and 1 M LiPF_6_, with the computed diffusion coefficients given in Table S7 alongside comparable values computed by Borodin and Smith^95^ (298 K). We find a ratio of Li^+^ to PF_6_^–^ diffusion coefficients of *D*_Li_/*D*_PF6_ = 0.60. Hayamizu^21^ found this ratio to be 0.57 for a similar electrolyte containing EC:DEC at 6:4 molar ratio and 1 M LiPF_6_ at 293 K (and a ratio of 0.59 at 303 K).

The trends of the EC-DMC and PC-DMC mixture permittivities are consistent with the saline ones—all permittivities are observed to increase as the EC and PC volume fraction is increased. Adding salt to systems with at least 25% EC or PC reduces their permittivity, a behavior which has also been observed and studied in saline water.^96^

**Figure 8:**
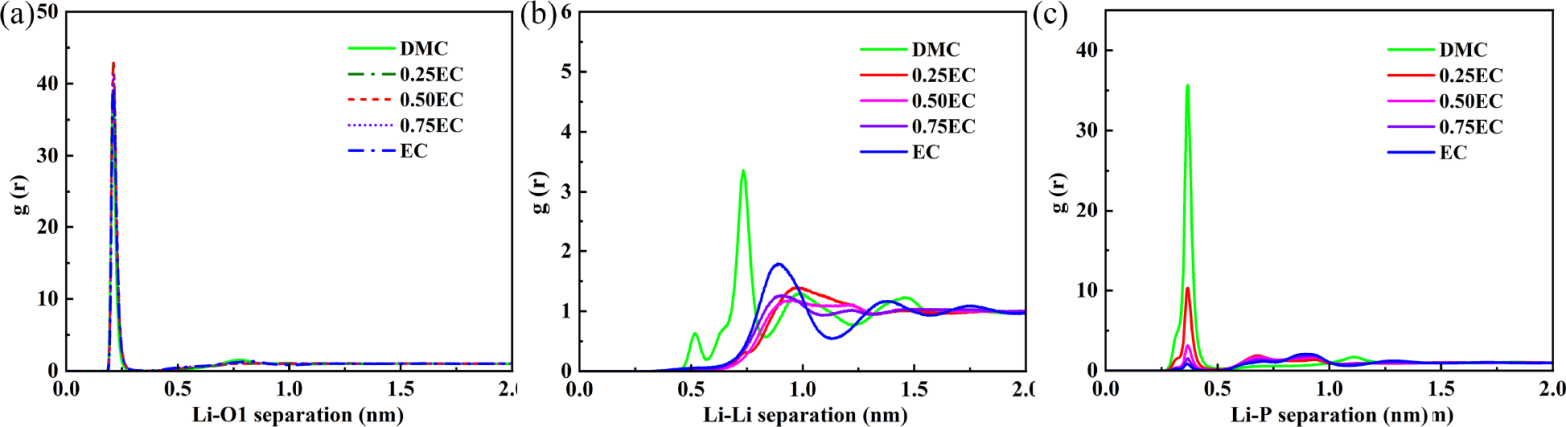
RDF, *g*(*r*), of Li^+^ ions with their surrounding O, Li^+^, and P atoms for varying EC volume fraction in the EC-DMC binary mixture with 1 M LiPF_6_ at 298 K: (a) Li–O; (b) Li–Li; (c) Li–P.

The RDF of the Li^+^ atoms with their surrounding O, Li^+^, and P atoms in mixed solvent (EC-DMC) is shown in Figure 8 to demonstrate how the ratio of high- and low-permittivity solvents affects the salt dissociation. An interesting transition is observed between 0% and 25% EC by volume, as the structure of the short-ranged part of the Li–Li RDF changes completely such that the first two peaks are no longer present at 25% EC and above. This corresponds to a break down of the ordered chain-like structures of the salt in pure DMC solvent seen in Figure 3g. As the EC volume percentage is increased to 100%, EC forms a complete solvation shell around the Li^+^ ions and therefore the peak of the Li–P RDF at 0.4 nm decreases close to zero.

Finally, we comment on the performance of the united-atom TraPPE model compared to a comparable all-atom model, in this case CHARMM. The improvement will depend primarily on the proportion of hydrogen atoms and choice of time step. When using a 1 fs time step, such as in refs (48 and 97), the relative performance of TraPPE (which uses a 2 fs time step) averaged over EC, PC, DMC, and DME simulations was found to be 3.6 times faster. However, commonly used techniques such as constraining C–H bonds to remove high-frequency motion allow the time step of all-atom simulations to be increased to 2 fs, in which case the speedup factor is 1.8.

## Conclusion

In this work, we have developed an extension to the TraPPE united-atom force field, compatible with widely used MD simulation codes including GROMACS, addressing the lack of efficient united-atom models validated for electrolyte solvents. This enables us to calculate important properties of electrolytes, such as self-diffusion coefficients, relative permittivity, surface tension, and viscosity more efficiently. A systematic procedure for computing the properties of binary electrolytes as a function of solvent composition is applied, enabling optimization of electrolyte formulations for utilization in electrochemical devices. We demonstrate how the transport properties of the electrolyte solution, comprised of LiPF_6_ salt and a binary mixture of two solvents, depends on the ratio of high- and low-permittivity solvents. Our results show that the LiPF_6_ salt is dispersed uniformly in pure EC and PC solvents, and in both cases we observe the formation of complete solvation shells around the Li^+^ ions, with six solvent molecules packed tightly. In contrast, LiPF_6_ in DMC formed linear chains of alternating charge ions, and in DME solvent the salt formed globular clusters. Finally, we find the Li^+^ and PF_6_^–^ diffusion coefficients are nearly identical in pure DMC solvent but deviate as EC is added, with the Li^+^ diffusion coefficient decreasing monotonically with increasing EC concentration.

## Data Availability

The most up to date force field files can be downloaded from the Github repository: https://github.com/SB8/trappe-electrolyte.

## Supporting Information Available

Description of dihedral potential optimization; plots of dihedral potential energy surfaces, dependence of viscosity on cosine-acceleration parameter, DMC conformer equilibration; optimized charges vs *r*_*p*_ parameter, and diffusion coefficient and permittivity dependences on *r*_*p*_; tables listing LJ parameters, force field parameters, computed properties, number of molecules in simulated systems, and diffusion coefficients (PDF)
Archive containing GROMACS force field implementation (ZIP)
