# Supplementary material for: Extension of the TraPPE Force Field for Battery Electrolyte Solvents
Source: J Phys Chem B. 2023 Mar 2;127(10):2224–36. doi: 10.1021/acs.jpcb.2c06993 (PMC10026065; doi:10.1021/acs.jpcb.2c06993)
Supplement: Supplementary file 1 — jp2c06993_si_001.pdf [file jp2c06993_si_001.pdf]

Supporting Information: Extension of the TraPPE force field for battery electrolyte solvents

Zhifen Luo,[†] Stephen A. Burrows,[‡] Stoyan K. Smoukov,[*,‡] Xiaoli Fan,[*,†] and Edo S. Boek[*,‡]

†*State Key Laboratory of Solidification Processing, School of Materials Science and Engineering, Northwestern Polytechnical University, 127 West Youyi Road, Xi'an Shaanxi, 710072, P.R.China*

‡*Chemical Engineering and Renewable Energy, School of Engineering and Materials Science, Queen Mary University of London, Mile End Road, London, E1 4NS, UK*

E-mail: s.smoukov@qmul.ac.uk; xlfan@nwpu.edu.cn; e.boek@qmul.ac.uk

Phone: +44 (0)20 7882 2964

Supporting Information

Force Field Repository

The most up to date force field files can be downloaded from the Github repository:

`https://github.com/SB8/trappe-electrolyte`

Dihedral Potential Optimization

The force field's dihedral (torsion) potentials are implemented in GROMACS using the cosine power series form,

$$V(\phi) = \sum_{n=0}^{5} C_n \left(\cos(\phi - \pi)\right)^n \ , \tag{1}$$

where V is the energy, ϕ is the dihedral angle using the convention that $\phi = 0$ for synperiplanar (cis) conformation, and the coefficients $\{C_n\}$ are the parameters to be optimized. As documented in Ref.,[S1] dihedral potential parameters are optimized by fitting to two-dimensional potential energy surfaces (PES) $U(\phi_1, \phi_2)$ where ϕ_1 and ϕ_2 are dihedral angles. The code developed in Ref.[S1] is hosted in the Github repository:

`https://github.com/SB8/Intra-Mol_Optimize`

Optimization is achieved by minimizing the objective function

$$F = \sum_i \sum_j \left[U_{DFT}(\phi_i, \phi_j) - U_{MD}(\phi_i, \phi_j)\right]^2 \exp\left(-\frac{U_{DFT} - U_0}{U_b}\right) \ . \tag{2}$$

This is the sum of the squared differences in the energy between the MD force field and the benchmark density functional theory (DFT) quantum chemistry data, which are weighted by a Boltzmann factor with parameter U_b, and with U_0 being the lowest energy in the PES. The purpose of this factor is to weight the points in the PES so that more emphasis is placed on the lower energy regions which are more probable geometries for the molecule. We choose a U_b value of 20.92 kJ/mol (5 kcal/mol) as was used in reference[S2] for Boltzmann-weighted fitting of dihedral potentials in di-peptides.

F is minimized using the descent-modified conjugate gradient algorithm proposed by Zhang.[S3] The descent direction at iteration k, $\mathbf{d}_k$, is given by

$$\mathbf{d}_k = -\mathbf{g}_k + \beta\mathbf{d}_{k-1} - \mu\left(\mathbf{g}_k - \mathbf{g}_{k-1}\right) \ , \tag{3}$$

where $\mathbf{g}$ is the gradient of F with respect to the parameters being optimized. i.e. each element of $\mathbf{g}$ is the derivative of F with respect to one of the dihedral coefficients C_i. The dependence of F on C_i is via the dihedral potential terms in U_{MD}. β and μ are scalar parameters defined as

$$\beta = \frac{\mathbf{g}_k \cdot (\mathbf{g}_k - \mathbf{g}_{k-1})}{|\mathbf{g}_{k-1}|^2} \quad \text{and} \quad \mu = \frac{\mathbf{g}_k \cdot \mathbf{d}_{k-1}}{|\mathbf{g}_{k-1}|^2} \ , \tag{4}$$

where $k-1$ denotes the previous iteration. Each step in the algorithm involves computing $\mathbf{d}_k$ and then performing a line search to satisfy the condition

$$F\left(\mathbf{x}_k + \alpha_d \mathbf{d}_k\right) \leq F\left(\mathbf{x}_k\right) - \delta \cdot \alpha_d^2 |\mathbf{d}_k|^2 \ , \tag{5}$$

where $\mathbf{x}$ are the current parameters, and α is a parameter which controls the distance of the line search along the descent direction. δ is a small parameter which can be set to zero, in which case the first point found to reduce F is accepted. α was set to unity, then successively reduced by a factor 0.5 until the condition of Eq. 5 is satisfied. The parameters are then updated as $\mathbf{x}_{k+1} = \mathbf{x}_k + \alpha_d \mathbf{d}_k$.

Figures S1–S4 show the dihedral angles chosen and the resulting PES, with corresponding MD fit and deviations. The dispersion-corrected B3LYP-D3 functional was used with the 6-311++G(2d,2p) basis set as cited in the main text.

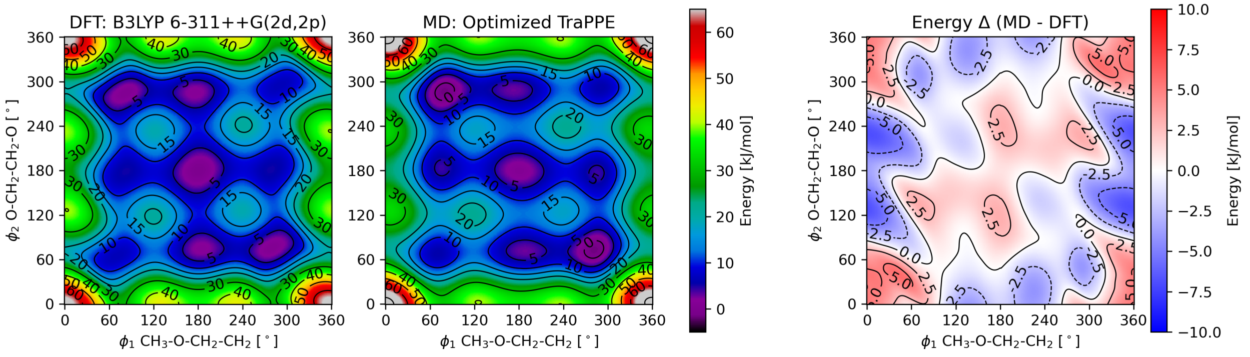

Figure S1: Comparison of DFT and TraPPE MD dihedral potential energy surface of DME.

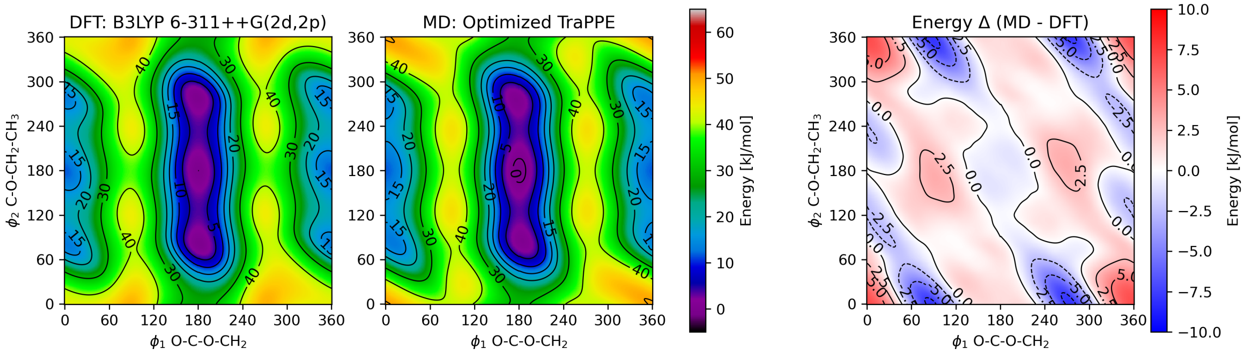

Figure S2: Comparison of DFT and TraPPE MD dihedral potential energy surface of DEC.

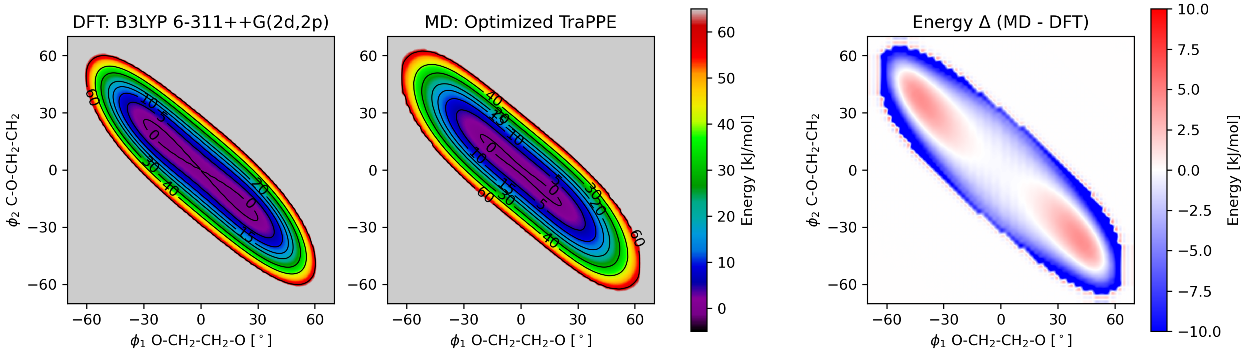

Figure S3: Comparison of DFT and TraPPE MD dihedral potential energy surface of EC.

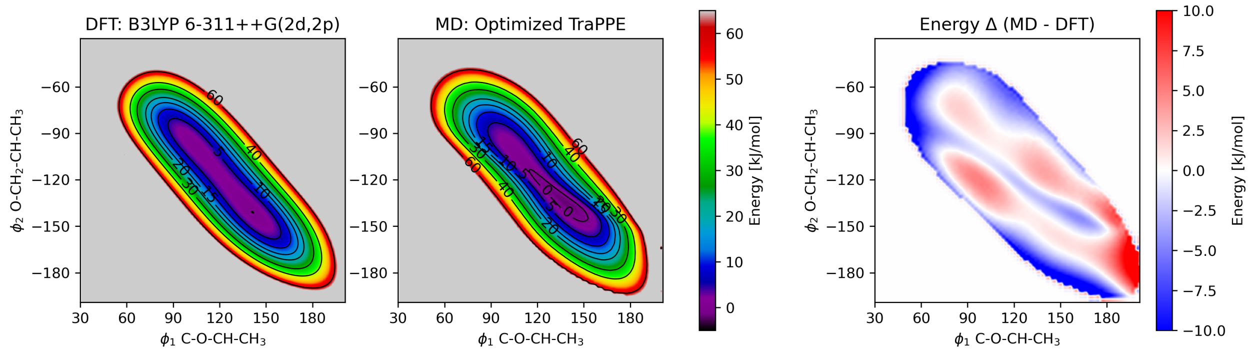

Figure S4: Comparison of DFT and TraPPE MD dihedral potential energy surface of PC.

Supplementary Figures and Tables

Table S1: $LiPF_6$ Lennard-Jones (LJ) parameters and charges.

Salt	Atom	σ (nm)	ϵ (kJ/mol)	Charge (|e|)
	Li^+	0.2126	0.0765	1.00
$LiPF_6$	P	0.3740	0.8368	1.34
	F	0.3118	0.2552	-0.39

Table S2: LJ parameters from the TraPPE force field[S4–S6] using TraPPE units (Å and K), with newly optimized charges.

Molecule	Atom	σ (Å)	ϵ/k_B (K)	Charge (|e|)
EC	O1	3.05	79.0	-0.547
	C2	3.82	40.0	0.825
	O3, O4†	2.20	190.0	-0.399
	C5, C6 (CH_2)†	3.88	56.3	0.260
PC	O1	3.05	79.0	-0.547
	C2	3.82	40.0	0.825
	O3, O4†	2.20	190.0	-0.399
	C5 (CH_2)†	3.88	56.3	0.260
	C6 (CH)	4.33	10.0	0.260
	C7 (CH_3)	3.75	98.0	0.0
DMC	O1	3.05	79.0	-0.614
	C2	3.82	40.0	0.932
	O3, O4	2.80	55.0	-0.448
	C5, C6 (CH_3)	3.75	98.0	0.289
DEC	O1	3.05	79.0	-0.614
	C2	3.82	40.0	0.932
	O3, O4	2.80	55.0	-0.448
	C5, C6 (CH_2)	3.95	46.0	0.289
	C7, C8 (CH_3)	3.75	98.0	0.0
DME	C1, C6 (CH_3)	3.75	98.0	0.204
	O2, O5	2.80	55.0	-0.428
	C3, C4 (CH_2)	3.95	46.0	0.224

†LJ parameters from Ref.[S6] (5-membered cyclic ether). Others from Ref.[S4,S5]

Table S3: LJ parameters and charges from the CHARMM-SwissParam (SP) and CHARMM36 force fields.

				Charge	
Molecule	Atom	σ (nm)	ϵ (kJ/mol)	CHARMM-SP (|e|)	CHARMM36† (|e|)
EC	O1	0.3029	0.5021	-0.547	-0.390
	C2	0.3563	0.2929	0.870	0.230
	O3, O4	0.2940	0.4184	-0.430	-0.240
	C5, C6 (CH_2)	0.3581	0.2343	0.280	0.140
	H (CH_2)	0.2388	0.1464	0.000	0.090
PC	O1	0.3029	0.5021	-0.547	-0.390
	C2	0.3563	0.2929	0.870	0.230
	O3, O4	0.2940	0.4184	-0.430	-0.240
	C5 (CH_2)	0.3581	0.2343	0.280	0.185
	H (CH_2)	0.2388	0.1464	0.000	0.090
	C6 (CH)	0.3564	0.1339	0.280	0.185
	H (CH)	0.2388	0.1883	0.000	0.090
	C7 (CH_3)	0.3653	0.3264	0.000	-0.270
	H (CH_3)	0.2388	0.1004	0.000	0.090
DMC	O1	0.3029	0.50208	-0.570	-0.390
	C2	0.3563	0.29288	0.870	0.230
	O3, O4	0.2940	0.4184	-0.43	-0.240
	C5, C6 (CH_3)	0.3653	0.3264	0.280	0.050
	H (CH_3)	0.2388	0.1004	0.000	0.090
DEC	O1	0.3029	0.5021	-0.570	-0.390
	C2	0.3563	0.2929	0.870	0.230
	O3, O4	0.2940	0.4184	-0.430	-0.240
	C5, C6 (CH_2)	0.3581	0.2343	0.280	0.140
	H (CH_2)	0.2388	0.1464	0.000	0.090
	C7, C8 (CH_3)	0.3653	0.3264	0.000	-0.270
	H (CH_3)	0.2388	0.1004	0.000	0.090
DME	C1, C6 (CH_3)	0.3653	0.3264	0.280	-0.100
	H (CH_3)	0.2388	0.1004	0.000	0.090
	O2, O5	0.2940	0.4184	-0.560	-0.340
	C3, C4 (CH_2)	0.3581	0.2343	0.280	-0.010
	H (CH_2)	0.2388	0.1464	0.000	0.090

†CHARMM36 parameters are obtained from CGenFF topology file (top_all36_cgenff.rtf).

Table S4: Molecule numbers of single solvent and salt systems.

Molecule	Num. of solvent	Num. of $LiPF_6$
EC	4620	308
PC	3623	308
DME	2839	308
DEC	2544	308
DMC	3627	308

Table S5: Molecule numbers of mixed solvent and salt systems.

Molecule	Vol. fraction of EC or PC	Num. of EC or PC	Num. of DMC	Num. of $LiPF_6$
EC-DMC	0.00	0	3627	308
	0.25	1155	2720	308
	0.50	2310	1814	308
	0.75	3465	907	308
	1.00	4620	0	308
PC-DMC	0.00	0	3627	308
	0.25	906	2720	308
	0.50	1812	1814	308
	0.75	2717	907	308
	1.00	3623	0	308

Table S6: Computed density (ρ), diffusion coefficient (D) and relative permittivity (ε) of EC (313 K), PC (303 K), DMC (303 K), DEC (298K) and DME (303 K) using the CHARMM-SP force field.

	solvent	computed value ± SD	experimental data	relative errors^h (%)
ρ (g/cc)	EC	1.247^g	1.323^a	-5.74
	PC	1.142	1.200^b	-4.79
	DMC	1.005	1.063^b	-5.45
	DEC	0.947	0.980^c	-3.34
	DME	0.878	0.860^b	2.06
D (10^{-10} m^2/s)	EC	5.561 ± 0.028	8.00^d	-30.49
	PC	4.672 ± 0.154	5.80^d	-19.44
	DMC	24.665 ± 0.262	26.00^d	-5.13
	DEC	11.645 ± 0.245	–	–
	DME	22.799 ± 1.185	31.00^d	-26.45
ε	EC	72.925 ± 4.189	89.00^e	-18.06
	PC	52.789 ± 1.184	64.90^f	-18.66
	DMC	1.186 ± 0.002	3.10^f	-61.74
	DEC	1.168 ± 0.002	2.80^c	-58.29
	DME	1.337 ± 0.064	7.20^f	-81.43

Experimental data: ^{a}from Ref.[S10], ^{b}Ref.[S11], ^{c}Ref.[S12], ^{d}Ref.[S8], ^{e}Ref.[S10], ^{f}Ref.[S11]
^{g}For ρ, all standard deviations (SD) are $< 10^{-4}$ g/cc and therefore not shown.
^{h}Relative errors are in comparison to experimental values.

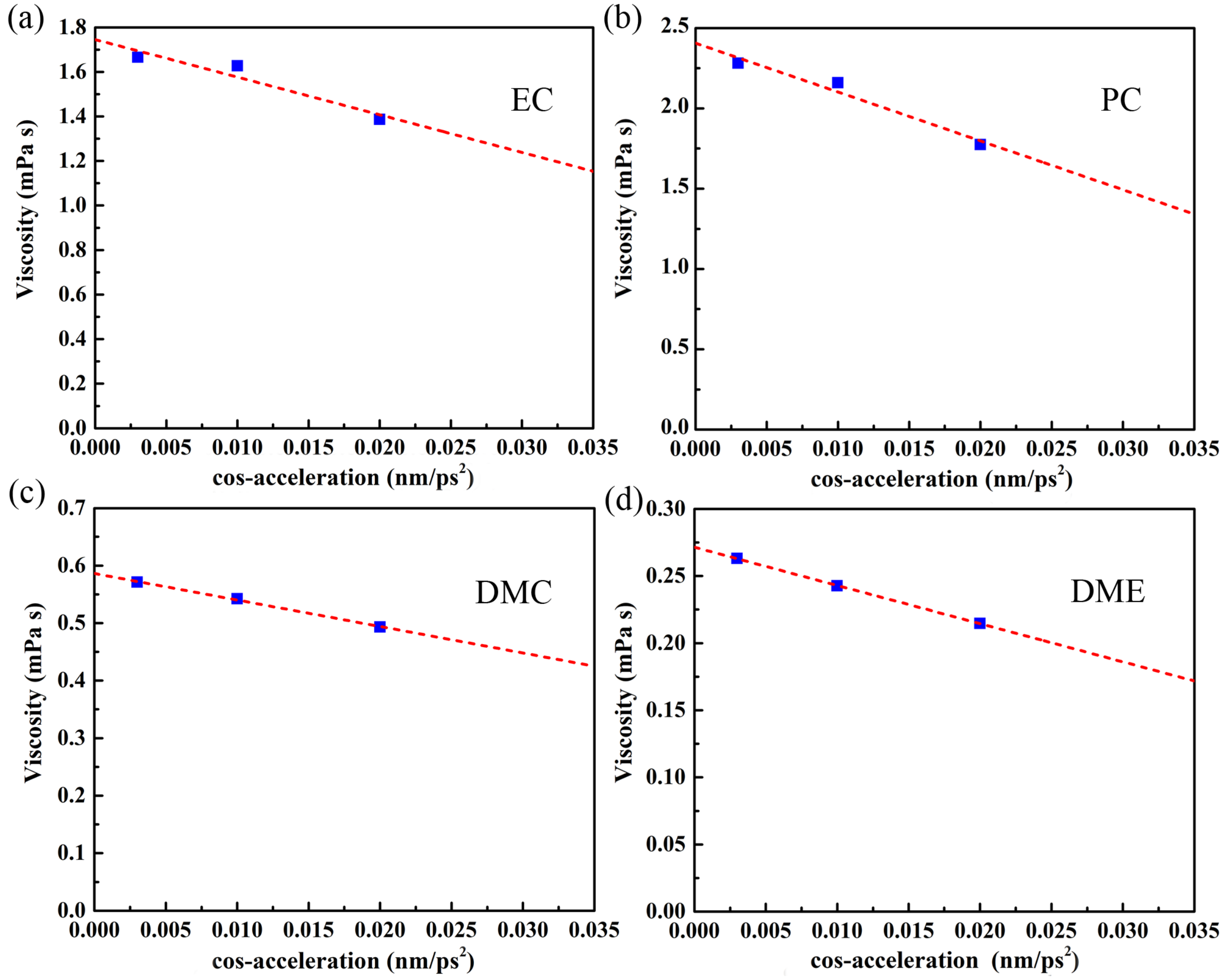

Figure S5: Dependence of calculated viscosity on the value of cos-acceleration for (a) EC, (b) PC, (c) DMC and (d) DEC.

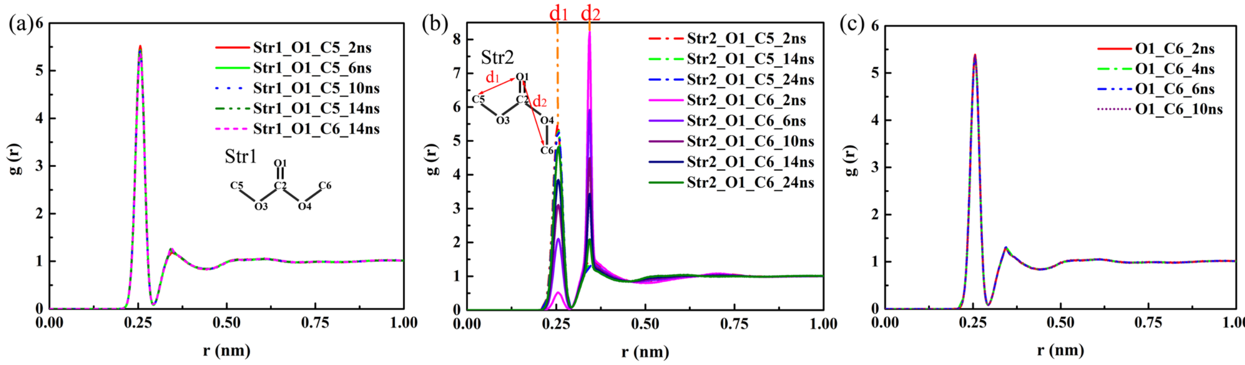

Figure S6: The RDF of O1 atoms (carbonyl oxygen) with intramolecular C5 and C6 atoms (terminal methyl groups) for two different DMC starting conformers (Str1 and Str2). (a) The RDF evolution starting from Str1; (b) The RDF evolution starting from Str2; (c) The RDF evolution starting from 99% Str1 mixed with 1% Str2.

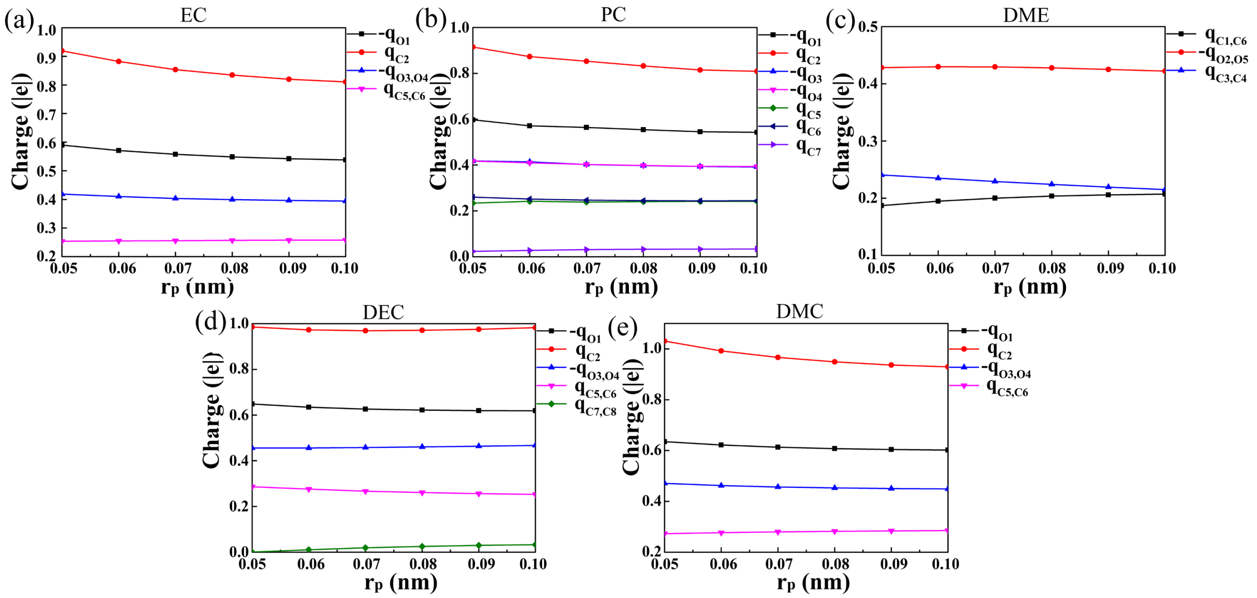

Figure S7: The optimized point charges, q, as a function of r_p for the molecules (a) EC, (b) PC, (c) DME, (d) DEC and (e) DMC. Oxygen atoms are all negatively charged and so $-q$ is plotted for them.

Table S7: Ions and solvent self-diffusion coefficients, D (10^{-10} m^2/s), for EC-DMC 50:50 wt% electrolyte with 1M $LiPF_6$ and ion charge scaling factor of 85% (298 K). Results are compared with simulation data in Fig. 10 of Ref.[S13]

	EC	DMC	Li^+	PF_6^-
this work	4.332	4.986	1.943	3.230
Ref.[S13]	6.64	7.29	2.49	2.90

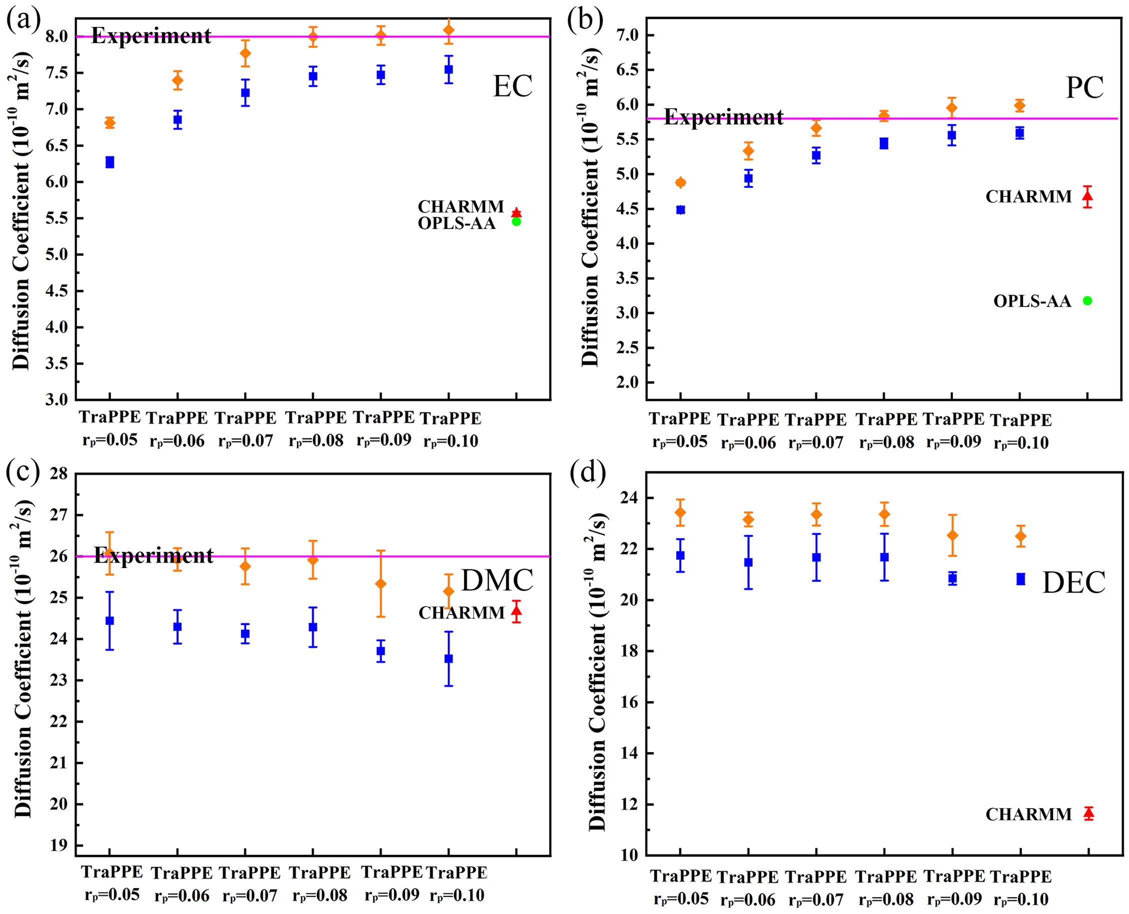

Figure S8: Computed self-diffusion coefficients of pure solvents (a) EC, (b) PC, (c) DMC and (d) DEC. OPLS-AA results for EC and PC are from Ref.,[S7] CHARMM-SP and TraPPE results are computed in this work. Horizontal pink lines are experimental results from Hayamizu *et al.*[S8] Blue squares are computed using the mean squared displacement method, and the orange diamonds after the finite-size correction of Yeh and Hummer[S9] is applied.

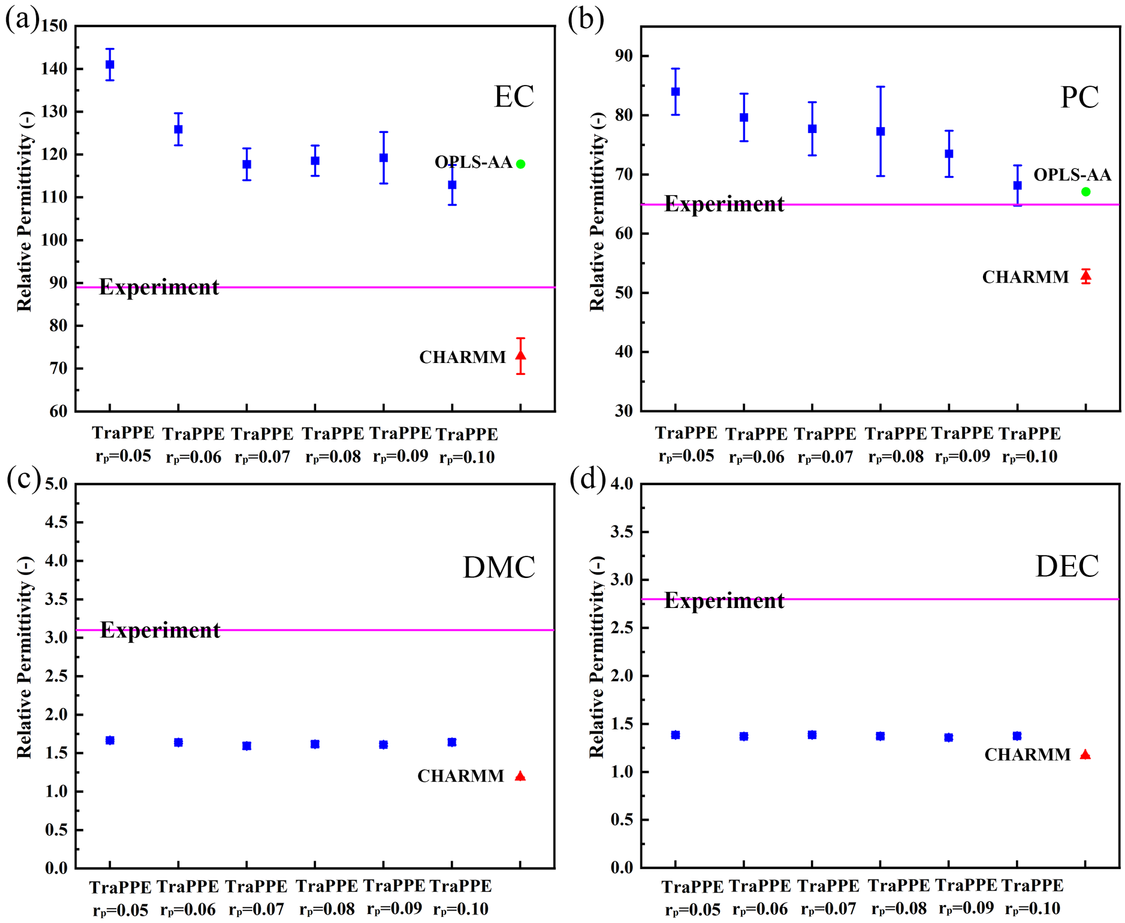

Figure S9: Computed relative permittivity of pure solvents (a) EC, (b) PC, (c) DMC and (d) DEC. OPLS-AA results for EC and PC are from Ref.,[S7] CHARMM-SP and TraPPE results are computed in this work. Horizontal pink lines represent the corresponding experimental results for EC,[S10] as well as PC, DMC and DEC.[S11]

References

(S1) Burrows, S. A. Multiscale simulation of transport phenomena in semi-solid flow batteries. Ph.D. thesis, Imperial College London, 2017.

(S2) Duan, Y.; Wu, C.; Chowdhury, S.; Lee, M. C.; Xiong, G.; Zhang, W.; Yang, R.; Cieplak, P.; Luo, R.; Lee, T., et al. A point-charge force field for molecular mechanics simulations of proteins based on condensed-phase quantum mechanical calculations. *Journal of Computational Chemistry* **2003**, *24*, 1999–2012.

(S3) Zhang, L.; Zhou, W.; Li, D.-H. A descent modified Polak–Ribière–Polyak conjugate gradient method and its global convergence. *IMA Journal of Numerical Analysis* **2006**, *26*, 629–640.

(S4) Stubbs, J. M.; Potoff, J. J.; Siepmann, J. I. Transferable potentials for phase equilibria. 6. United-atom description for ethers, glycols, ketones, and aldehydes. *The Journal of Physical Chemistry B* **2004**, *108*, 17596–17605.

(S5) Maerzke, K. A.; Schultz, N. E.; Ross, R. B.; Siepmann, J. I. TraPPE-UA force field for acrylates and Monte Carlo simulations for their mixtures with alkanes and alcohols. *The Journal of Physical Chemistry B* **2009**, *113*, 6415–6425.

(S6) Keasler, S. J.; Charan, S. M.; Wick, C. D.; Economou, I. G.; Siepmann, J. I. Transferable potentials for phase equilibria–united atom description of five-and six-membered cyclic alkanes and ethers. *The Journal of Physical Chemistry B* **2012**, *116*, 11234–11246.

(S7) You, X.; Chaudhari, M. I.; Rempe, S. B.; Pratt, L. R. Dielectric relaxation of ethylene carbonate and propylene carbonate from molecular dynamics simulations. *The Journal of Physical Chemistry B* **2016**, *120*, 1849–1853.

(S8) Hayamizu, K.; Aihara, Y.; Arai, S.; Martinez, C. G. Pulse-gradient spin-echo ^{1}H, ^{7}Li, and ^{19}F NMR diffusion and ionic conductivity measurements of 14 organic electrolytes containing LiN $(SO_2CF_3)_2$. *The Journal of Physical Chemistry B* **1999**, *103*, 519–524.

(S9) Yeh, I.-C.; Hummer, G. System-size dependence of diffusion coefficients and viscosities from molecular dynamics simulations with periodic boundary conditions. *The Journal of Physical Chemistry B* **2004**, *108*, 15873–15879.

(S10) Naejus, R.; Damas, C.; Lemordant, D.; Coudert, R.; Willmann, P. Excess thermodynamic properties of the ethylene carbonate–trifluoroethyl methyl carbonate and propylene carbonate–trifluoroethyl methyl carbonate systems at T=(298.15 or 315.15) K. *The Journal of Chemical Thermodynamics* **2002**, *34*, 795–806.

(S11) Xu, K. Nonaqueous liquid electrolytes for lithium-based rechargeable batteries. *Chemical Reviews* **2004**, *104*, 4303–4418.

(S12) Uchida, S.; Kiyobayashi, T. What differentiates the transport properties of lithium electrolyte in ethylene carbonate mixed with diethylcarbonate from those mixed with dimethylcarbonate? *Journal of Power Sources* **2021**, *511*, 230423.

(S13) Borodin, O.; Smith, G. D. Quantum chemistry and molecular dynamics simulation study of dimethyl carbonate: ethylene carbonate electrolytes doped with $LiPF_6$. *The Journal of Physical Chemistry B* **2009**, *113*, 1763–1776.
